# Novel polymer micelle mediated co-delivery of doxorubicin and P-glycoprotein siRNA for reversal of multidrug resistance and synergistic tumor therapy

**DOI:** 10.1038/srep23859

**Published:** 2016-03-31

**Authors:** Chun-ge Zhang, Wen-jing Zhu, Yang Liu, Zhi-qiang Yuan, Shu-di Yang, Wei-liang Chen, Ji-zhao Li, Xiao-feng Zhou, Chun Liu, Xue-nong Zhang

**Affiliations:** 1 Department of Pharmaceutics, College of Pharmaceutical Sciences, Soochow University, Suzhou 215123, People’s Republic of China; 2 The first affiliated hospital of Soochow university, Suzhou 215123, People’s Republic of China; 3 College of Radiological Medicine and Protection, Soochow University, Suzhou 215123, People’s Republic of China; 4 Changshu Hospital of Traditional Chinese Medicine, Changshu 215500, People’s Republic of China; 5 The hospital of Suzhou People’s Hospital affiliated to Nanjing Medical University, Suzhou, 215000, People’s Republic of China

## Abstract

Co-delivery of chemotherapeutics and siRNA with different mechanisms in a single system is a promising strategy for effective cancer therapy with synergistic effects. In this study, a triblock copolymer micelle was prepared based on the polymer of N-succinyl chitosan–poly-L-lysine–palmitic acid (NSC–PLL–PA) to co-deliver doxorubicin (Dox) and siRNA–P-glycoprotein (P-gp) (Dox–siRNA-micelle). Dox–siRNA-micelle was unstable in pH 5.3 medium than in pH 7.4 medium, which corresponded with the *in vitro* rapid release of Dox and siRNA in acidic environments. The antitumor efficacy of Dox–siRNA-micelle *in vitro* significantly increased, especially in HepG2/ADM cells, which was due to the downregulation of P-gp. Moreover, almost all the Dox–siRNA-micelles accumulated in the tumor region beyond 24 h post-injection, and the co-delivery system significantly inhibited tumor growth with synergistic effects *in vivo*. This study demonstrated the effectiveness of Dox–siRNA-micelles in tumor-targeting and MDR reversal, and provided a promising strategy to develop a co-delivery system with synergistic effects for combined cancer therapy.

Combination therapy and multidrug resistance (MDR) reversal are hot issues for improving therapeutic effects and reducing side effects^1^,^2^,^3^,^4^. Simultaneous co-delivery of chemotherapeutics and siRNA in a single vehicle in cancer chemotherapy is more effective than co-treatment with either chemotherapeutics or siRNA^5^,^6^,^7^,^8^. MDR plays an important role in limiting the effective treatment of cancer^9^,^10^,^11^. P-glycoprotein (P-gp), one of the major energy-dependent efflux transporters that contribute to MDR, is encoded by the *mdr1* gene, thereby attenuating the efficiency of treatment in drug-resistant tumors by decreasing intracellular concentrations of chemotherapeutic agents^12^,^13^. A novel approach for reversing MDR is to downregulate the expression of P-gp using RNA interference^14^.

The clinical applications of chemotherapeutics are facing a series of challenges that hinder the effectiveness of chemotherapy, including insolubility (making them difficult to administer), non-targeting (causes insufficient penetration to tumors), and MDR (decreases intracellular accumulation)^15^. Polymeric micelles provide insight into overcoming the limitations of chemotherapeutics by increasing solubility and decreasing acute toxicity in healthy tissues. Encapsulating chemotherapeutics into micelle nanoparticles can bypass the efflux pumps and increase the intracellular accumulation of chemotherapeutics^16^,^17^.

siRNA delivery also faces tremendous barriers before accumulating in the targeted cytoplasm, including negative phosphate charges and large molecular weight (making them difficult to cross cellular membranes), short half-life in blood (rapidly degraded by nucleases), and poor cellular uptake (decreases intracellular accumulation); these limitations decrease the effectiveness of therapy^18^,^19^,^20^. Encapsulating siRNA into nanoparticles can prevent RNase degradation and renal clearance, and increase its half-life in the bloodstream^21^,^22^. Polymeric micelles based on synthetic or natural cationic polymers, such as polyethyleneimine^8^, poly-L-lysine (PLL)^23^, and chitosan^24^, have great advantages in chemical modification, physiological stability, and biological safety as gene or siRNA carriers over cationic lipids.

Although delivery systems carrying either chemotherapeutics or siRNA are effective in the co-treatment of cancer^25^,^26^,^27^,^28^, the combination of siRNA-based therapy with traditional chemotherapy in the same delivery system is more beneficial^29^. In the present study, we developed a co-delivery system based on the polymer of N-succinyl chitosan–PLL–palmitic acid (NSC–PLL–PA). NSC, the hydrophilic shell, was designed to increase the half-life of micelle and decrease the toxicity of PLL. PLL, the cationic backbone, was expected to electrostaticaly absorb the negatively charged siRNA. PA, the hydrophobic core, was used to encapsulate Dox. The triblock polymer micelle co-delivering Dox and siRNA (Dox–siRNA-micelle) was designed to downregulate P-gp expression, overcome MDR, and exert synergistic therapeutic effects (Fig. 1). The properties of micelles were characterized, and the ability to simultaneously deliver Dox and siRNA-P-gp was examined. Cellular uptake and subcellular localization characteristics were also investigated, and their tumor-targeting, antitumor, and antidrug-resistance properties were further confirmed.

## Materials and Methods

### Materials, cell lines, and tumor models

Doxorubicin hydrochloride (Dox-HCl) was supplied by Dalian Meilun Biotech Co., Ltd. (Dalian, China). NSC–PLL–PA triblock copolymer was synthesized in our laboratory^30^. Spectra Multicolor Broad Range Protein Ladder was purchased from Thermo Fisher Scientific (MA, USA). Anti-P-glycoprotein mouse mAb (C219) was purchased from Calbiochem (Darmstadt, Germany). Anti-mouse IgG (H+L) HAS labeled with Dylight 800 was purchased from KPL, Int. (MD, USA). Lipofectamine 2000 (Life Technologies Corporation, CA, USA) and Opti-MEM Reduced Serum Medium (Gibco, CA, USA) were used according to the manufacturer’s instructions. Targeting human P-gp siRNA (sense: 5′-GAAACCAACUGUUAGUGUAdTdT-3′; anti-sense: 5′-UACACUGACAGUUGGUUUCdTdT-3′), negative control siRNA (NC-siRNA), and fluorescein-labeled siRNA (FAM-siRNA) were supplied by Shanghai GenePharma Co. Ltd. (Shanghai, China). All other materials were used without further treatment.

HepG2 human liver cancer cells were cultured in RPMI 1640 medium containing 10% fetal bovine serum (FBS). HepG2/ADM cells with P-gp overexpression were cultivated in DMEM containing 10% FBS and 1% penicillin/streptomycin. All cells were cultured at 37 °C with 5% CO_2_ before use. The inoculated density was 5 × 10^4^ cells/well for a six-well plate and 5 × 10^3^ cells/well for a 96-well plate.

Female nude mice (four weeks old) were supplied by Shanghai SLRC Laboratory Animal Company (Shanghai, China). All animals were fed in accordance with the National Institutes of Health guidelines, and the procedures were performed consistent with the requirements of the Institutional Animal Care and Use Committee. All experimental proocol were approved by Medical ethics committee of Soochow University.

To establish the subcutaneous tumor model, HepG2/ADM cells (1 × 10^7^) or HepG2/ADM cells (1 × 10^7^) were subcutaneously injected into the armpit of nude mice. The liver tumor model *in situ* was established by orthotopic inoculation using the method described by our previous report^31^, and tumor-bearing nude mice were used four weeks post-administration.

### Preparation and evaluation of Dox–siRNA-micelle

Blank NSC–PLL–PA micelles (blank-micelle) were prepared by directly adding NSC–PLL–PA copolymers to distilled water with a concentration of 1 mg·mL^−1^ and then treated with ultrasonication for 10 min in an ice bath.

Dox-loaded NSC–PLL–PA micelles (Dox-micelle) were prepared as follows: 1 mg of Dox·HCl was dissolved into 500 μL of DMSO solution and then deprotonated by adding 0.5 μL of triethylamine to obtain hydrophobic Dox. About 500 μL of Dox–DMSO solution was added dropwise to blank-micelles (5 mL) under ultrasonic agitation. The mixture was stirred for 30 min and dialyzed against water for 4 h to remove the organic solvent DMSO and free Dox.

SiRNA-loaded NSC–PLL–PA micelles (siRNA-micelle) were prepared as follows: blank-micelles were prepared and mixed with a predetermined amount of siRNA (siRNA–P-gp or NC–siRNA) by gentle pipetting. The mixture was kept still for 20 min to form a micelleplex before use. FAM-labeled siRNA-micelles (siRNA ^FAM^-micelle) were prepared similarly.

NSC–PLL–PA micelles for co-delivery of Dox and siRNA (Dox–siRNA-micelle) were prepared by mixing Dox-micelle with a desired amount of siRNA in the same manner as described above.

The sizes and morphology of the Dox–siRNA-micelle were investigated by transmission electron microscopy (TEM) (FEI, Hillsboro, OR, USA), atomic force microscopy (AFM) (Bruker, Billerica, MA, USA), and Zetasizer Nano ZS (Malvern Instrument, USA).

### Stability of Dox–siRNA-micelle

To evaluate the stability of Dox–siRNA-micelle, the micelleplex was diluted with PBS at different pH values (7.4 and 5.3). The change in the size and zeta potential was examined by Zetasizer Nano ZS after incubation at various time points.

### Cellular uptake and subcellular localization of Dox–siRNA-micelle

Confocal laser scanning microscopy (CLSM) (Leica TCS SP5, Germany) was adopted to image the subcellular location of triblock polymer micelles. Cover slips were placed into a six-well plate. HepG2/ADM cells were seeded into the six-well plate and incubated for 24 h. The cells were each treated with Dox-micelle (5 μg·mL^−1^), siRNA^FAM^-micelle (50 nM) or Dox–siRNA^FAM^-micelle (5 μg·mL^−1^ equivalent Dox and 50 nM siRNA) for 1 h. After incubation, the cells were stained with Hoechest 33342 (10 μg·mL^−1^) for 10 min, fixed with paraformaldehyde (4% in PBS) for 10 min, washed twice with cold PBS, and imaged via CLSM.

A live cell station (Cell’ R, Olympus, Japan) was used to observe the dynamic process of cellular uptake and subcellular location. HepG2 and HepG2/ADM cells were each cultured in a glass bottom cell culture dish for 24 h, stained with Hoechest 33342 (10 μg·mL^−1^) for 10 min, and incubated with Dox–siRNA^FAM^-micelle (5 μg·mL^−1^ equivalent Dox and 50 nM siRNA) for detection. The uptake images of Dox–siRNA^FAM^-micelle within 3 h after administration were observed using the live cell station.

FACSCalibur (BD, USA) was used to analyze the characteristics of cellular uptake. HepG2/ADM cells in the six-well plate were treated with Dox–siRNA^FAM^-micelle (5 μg·mL^−1^ equivalent Dox and 50 nM siRNA) for 0.5, 1, 2, 4, or 6 h. The cells were washed three times with cold PBS, trypsinized, collected, and resuspended in 400 μL of PBS. Dual-color FACSCalibur was adopted to analyze the fluorescence intensity with FL1 band-pass for siRNA^FAM^ and FL2 band-pass for Dox. Cells incubated with Dox-micelle (5 μg·mL^−1^) or siRNA^FAM^-micelle (50 nM) for 6 h were used to adjust fluorescent compensation, and the untreated cells were used as the control in dual-color flow cytometry.

To quantitatively investigate intracellular drug uptake and elimination, HepG2 and HepG2/ADM cells in the six-well plate were each treated with Dox, Dox-micelle, or Dox–siRNA-micelle (20 μg·mL^−1^ equivalent Dox and 50 nM siRNA) for different times (0, 5, 15, 30, 60, 120, 240, and 360 min). Cells were then washed three times with cold PBS, scraped, and subjected to ultrasonic cell disruption. The intracellular Dox concentration and protein content in each well were measured by fluorescence spectrophotometry and the Coomassie Brilliant Blue method. Considering that the extracellular Dox concentration was much higher than the intracellular Dox concentration, Dox formulations were assumed to uptake at a constant speed (*K*_0_) and eliminate at a rate constant (*K*). The intracellular dynamics model was illustrated as previously reported^32^. *K*_0_ and *K* were obtained by non-linear fitting using Origin software.

### Cell viability assay

To evaluate the effect of siRNA concentration on cell viability, the cytotoxicity of Dox–siRNA-micelle with various siRNA concentrations was detected via MTT assay. HepG2/ADM cells in 96-well plates were treated with Dox–siRNA-micelle containing different concentrations of siRNA (25, 50, 100, 150, and 200 nM) for 24 h. About 20 μL of MTT (5 mg·mL^−1^ in PBS) was added into each well and further treated for 4 h. The medium was replaced with 150 μL of DMSO and gently shaken for 5 min. The absorbance at 570 nm was recorded by a microplate reader. The cell viability rate (VR) was calculated as follows:


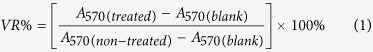


where A_570nm (treated)_ is the absorbance of wells treated with samples, A_570nm (non-treated)_ is the absorbance of non-treated wells, and A_570nm (blank)_ is the absorbance of blank wells.

The cytotoxicity of different Dox formulations in HepG2 and HepG2/ADM cells was also evaluated by MTT assay. Two types of cells in 96-well plates were each treated with different Dox formulations (including Dox, Dox-micelles, and Dox–siRNA-micelles with different Dox concentrations, constant siRNA content, N/P ratio of 20) for 24 or 48 h. The cells treated with equal amounts of blank-micelle were set as the controls. After incubation, the cells were treated as described above, and the half maximal inhibitory concentration (IC_50_) of different Dox formulations was calculated.

### Intracellular accumulation of Dox

To investigate the relationship between cytotoxicity and intracellular accumulation of chemotherapeutic agents, HepG2 and HepG2/ADM cells in a six-well plate were each incubated with Dox (5 μg·mL^−1^), Dox-micelles (5 μg·mL^−1^), or Dox–siRNA-micelles (5 μg·mL^−1^ equivalent Dox and 100 nM siRNA) for 6 h. The cells were treated as described above and then analyzed by CLSM and flow cytometry.

### Micelle distribution in nude mice

Cy7-labeled NSC–PLL–PA was synthesized using our previously reported procedure^33^, and Cy7-labeled micelle (Cy7-micelle) was prepared using the method described above. All experiments were performed in the dark.

To investigate the tumor-targeting ability of micelles, nude mice with subcutaneous or *in situ* liver tumor were injected with Cy7-micelles via the tail vein. At preset time points (1, 6, 12, 24, 36, and 48 h) post-administration, the mice were narcotized and analyzed with an *in vivo* imaging system (IVIS Lumina II; CaliperLife Sciences, Hopkinton, MA, USA). The mice were sacrificed at the final time point, and the major organs were collected for imaging.

### *In vivo* antitumor activity

Tumor suppression was studied using a subcutaneous tumor model. Tumor-bearing nude mice pre-administered with HepG2/ADM cells were randomly divided into six groups (*n* = 5). The mice were injected with different formulations via the tail vein when the subcutaneous tumor volume reached approximately 30 mm^3^ [including normal saline, Dox, blank-micelle, siRNA-micelle, Dox-micelle, and siRNA–Dox-micelle (0.5 mg·kg^−1^ equivalent Dox and 0.2 mg·kg^−1^ equivalent siRNA)]. The mice were administered every 3 d within 24 d. Tumor size and weight of the tumor-bearing nude mice were measured after every administration. After the last administration, the tumor-bearing nude mice were sacrificed, and tumors were collected and imaged. The tumor tissues were weighed, cut into pieces, and lysed in RIPA lysis buffer (200 μL/20 mg tissues) including 1 mM PMSF for 30 min on ice. The lysates were treated as described above. Exactly 80 μg of total protein was analyzed using Western blot. The tumor volumes in the tumor-bearing nude mice after every injection were estimated by the following equation as reported previously^3^:


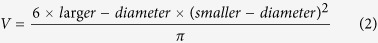


### Statistical analysis

Data are represented as the mean ± standard deviation of independent measurements. For statistical analysis between two groups, Student’s t-test for independent means was used. A value of P < 0.05 was considered as statistically significant, and a P-value less than 0.01 was considered as very significant. The differences between the overall therapeutic effects of different treatments were analyzed by one-way analysis of variance (ANOVA) with LDL multiple comparison test. Statistical analysis was performed using the SPSS software.

## Results and Discussions

### Preparation and evaluation of Dox–siRNA-micelle

Our previous report has showed that the encapsulation efficiency and drug loading of Dox-micelles were 95.32 ± 2.06% and 16.09 ± 0.17%, respectively. Meanwhile, siRNA was absorbed in the micelle through an electrostatic interaction with the PLL skeleton, and even compressed into the core to form the dense micelleplex. siRNA could efficiently bind to the micelle at an N/P ratio of 20/1 as determined by agarose gel electrophoresis^30^.

**Figure 2:**
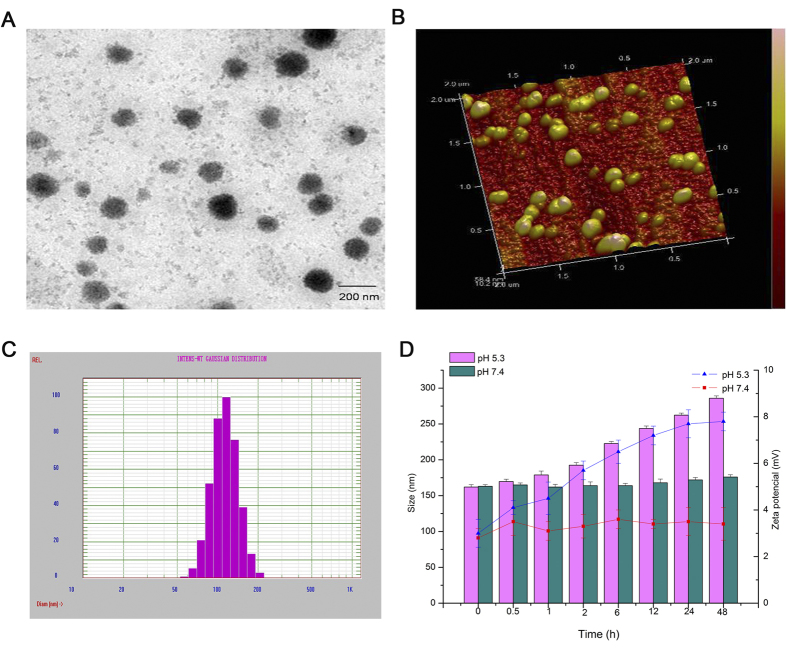
TEM micrographs (**A**), AFM micrographs (**B**), and particle diameter distribution (**C**) of siRNA–Dox-micelle. (**D**) Changes in size and zeta potential of Dox–siRNA-micelle in different pH media after incubation at various times (*n* = 3).

The particle size and zeta potential of micelles measured using Zetasizer Nano ZS were 179 nm and 3.2 mV, respectively, accompanied with a narrow size distribution and good dispersion (Fig. 2C). The size and surface morphology of micelles confirmed by TEM (Fig. 2A) and AFM (Fig. 2B) showed that most of the micelle nanoparticles were compact and spherical, with an average diameter of approximately 170 nm. This size was smaller than the result measured by Zetasizer Nano ZS, which was due to the collapse and shrinking that occurred during sample preparation for detection^34^.

### Stability of Dox–siRNA-micelle

The changes in size and zeta potential of Dox–siRNA-micelle after incubation with different pH buffer solutions for various times are shown in Fig. 2D. Dox–siRNA-micelle was quite stable in pH 7.4 PBS, but the particle size and zeta potential increased with increasing incubation time in pH 5.3 PBS. This behavior was assumed to be caused by the instability of micelles in pH 5.3 PBS. The hydrophilicity of triblock polymer increased due to the re-protonation of the amino group in low pH, whereas the PA block was too short to maintain micelle stability, resulting in sudden demicellization and rapid release of the entrapped drug^35^,^36^.

### Cellular uptake and subcellular location of Dox–siRNA-micelle

**Figure 3:**
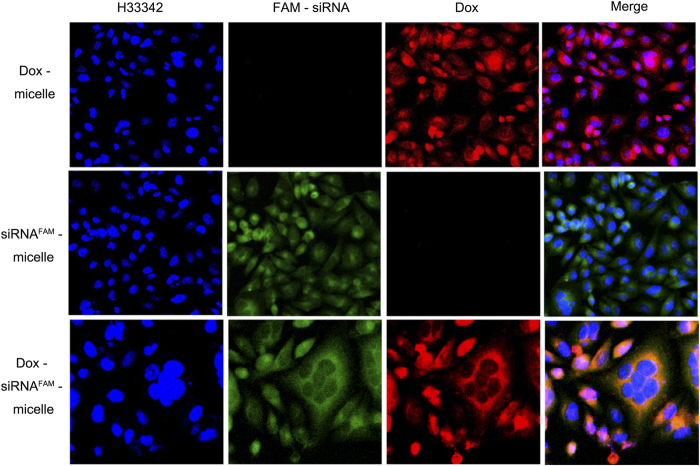
Subcellular localization of Dox-micelle, siRNA-micelle, and Dox–siRNA-micelle in HepG2/ADM cells evaluated by CLSM after incubation for 1 h.

**Figure 4:**
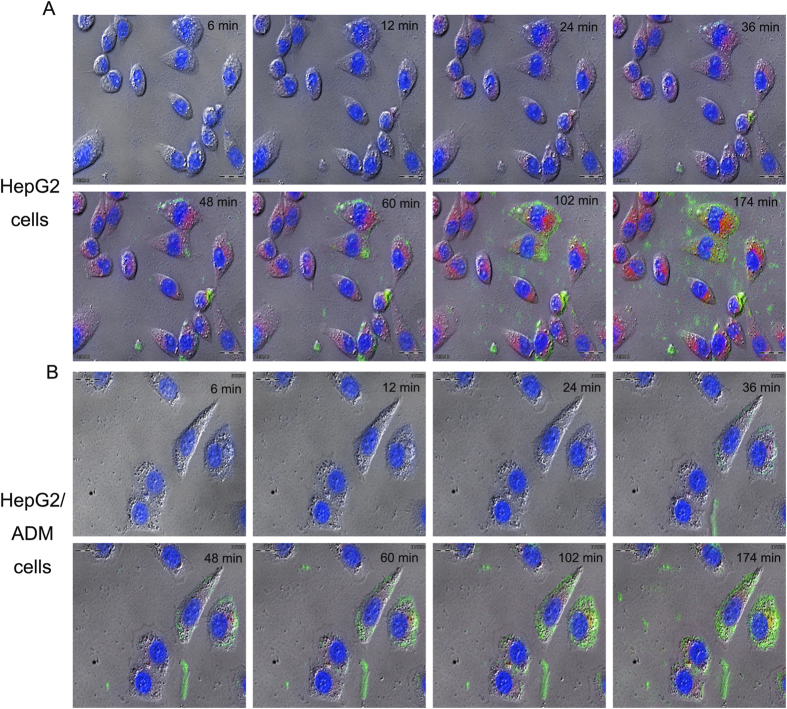
Uptake images of Dox–siRNA-micelle in HepG2 cells (**A**) or HepG2/ADM cells (**B**) within 3 h after administration observed using a live cell station.

Cellular uptake and intracellular distribution of Dox–siRNA-micelle in HepG2/ADM cells were measured by CLSM (Fig. 3). The co-localization of Dox and siRNA^FAM^ in the cytoplasm indicated the simultaneous delivery of siRNA^FAM^ and Dox in HepG2/ADM cells after incubation with Dox–siRNA^FAM^-micelle. The preponderant cellular uptake and intracellular release were further confirmed by a live cell station (Fig. 4). Dox and siRNA^FAM^ were simultaneously delivered by Dox–siRNA^FAM^-micelle in the cells and then released into the cytoplasm. Dox was transferred to the nucleus and embedded into the DNA bases, whereas siRNA stayed within the cytoplasm to disrupt mRNA transcription and downregulate the expression of the target protein.

**Figure 5:**
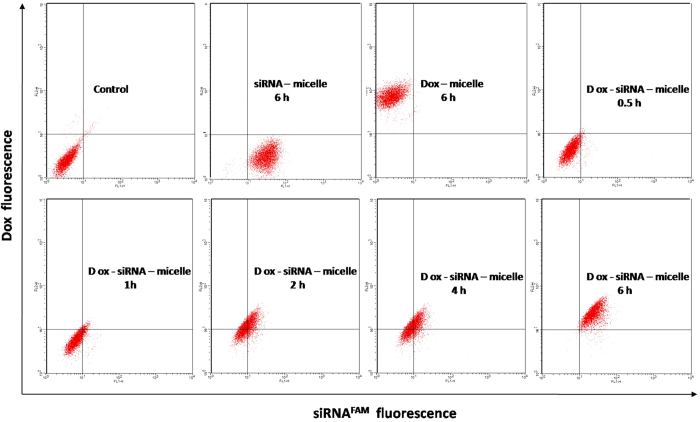
Detection of cellular uptake of Dox–siRNA^FAM^-micelle in HepG2/ADM cells within 6 h after administration using flow cytometry.

Cellular uptake and intracellular accumulation of Dox–siRNA-micelle in HepG2/ADM cells were also detected by two-color flow cytometry (Fig. 5). Both green- and red-positive cells were detected after incubation with Dox–siRNA^FAM^-micelle, and the proportion increased with the incubation time. This finding indicated that Dox–siRNA-micelle could simultaneously deliver siRNA and Dox to cells, resulting in co-localization in the same cancer cells. This finding was in accordance with the aforementioned observations.

**Figure 6:**
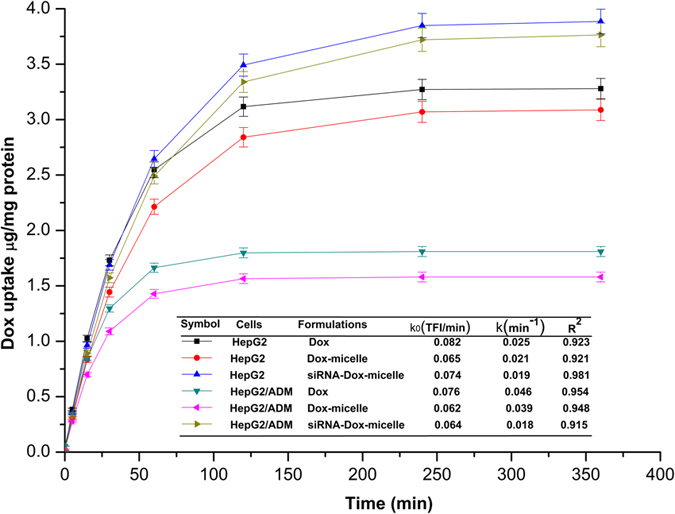
Intracellular drug accumulation of HepG2 or HepG2/ADM cells after treatment with Dox, Dox-micelle, and Dox–siRNA-micelle for different times. Drug uptake kinetic parameters obtained by non-linear fitting using Origin software (*n* = 3).

The dynamic changes in intracellular Dox amount after treatment with different Dox formulations were measured and non-linearly fitted to simulate cellular uptake kinetics (Fig. 6). The uptake rate of Dox was faster than that of micelles, but no significant difference was observed between Dox-micelle and Dox–siRNA^FAM^-micelle both in HepG2 and HepG2/ADM cells. This finding might be due to the different uptake pathways of Dox and micelles. Dox entered cells via diffusion depending on the concentration difference between extracellular and intracellular environments, whereas micelle uptake occurred via endocytosis depending on the related protein on the cytomembrane. The extracellular Dox concentration was much higher than the intracellular Dox concentration, whereas the amount of related protein on the cytomembrane was limited. The elimination rate constant of Dox was also larger than that of Dox-micelle, indicating that the release process of Dox-micelle was slow, whereas Dox, as the substrate of P-gp, was effluxed immediately. The elimination rate constant of Dox–siRNA-micelle in HepG2/ADM cells was smallest, which suggested that siRNA was released from micelles in the cytoplasm, disturbed the translation of mdr1 mRNA, and downregulated the expression of P-gp, resulting in decreased efflux.

### Cell viability assay

**Figure 7:**
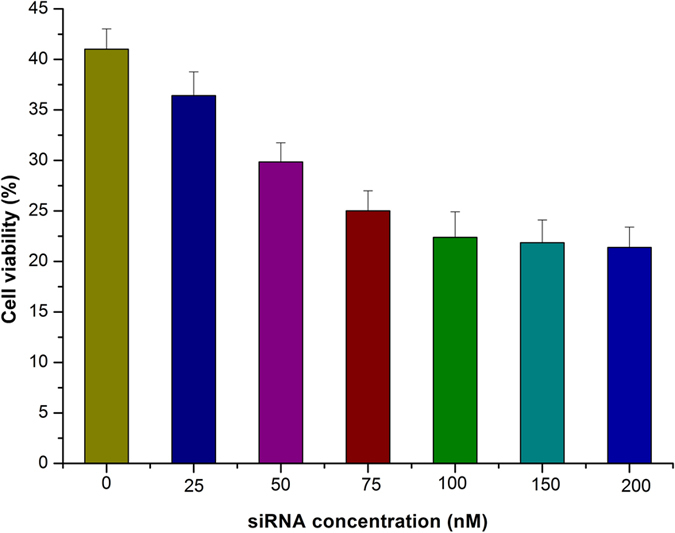
Cytotoxicity of Dox in HepG2/ADM cells after incubation with Dox–siRNA-micelle at various siRNA concentrations (**B**) (*n* = 3).

MTT assays were performed to confirm the potential antitumor efficacy of a co-delivery system combining chemotherapeutics and P-gp downregulation. The siRNA concentration in Dox–siRNA-micelle was screened to exert maximum therapeutic effects with the optimal combination of P-gp downregulation and chemotherapeutics (Fig. 7). The cytotoxicity of Dox–siRNA-micelle increased in a dose-dependent manner within a certain siRNA concentration range. By contrast, the therapeutic efficacy was close to the maximum platform when the siRNA concentration reached about 100 nM and did not improve with increasing concentration. This finding indicated that 100 nM was sufficient to downregulate P-gp expression, increase intracellular Dox concentration, and maximize the therapeutic effects.

**Figure 8:**
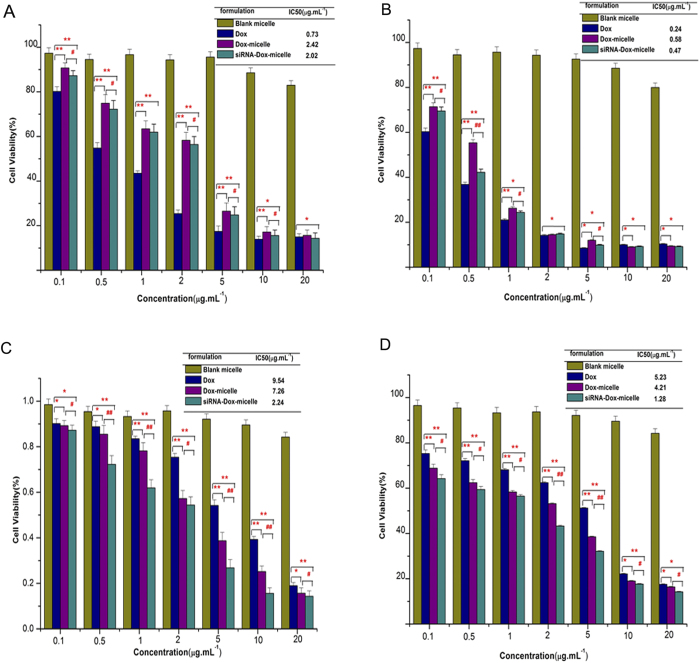
Cytotoxicity of different Dox formulations in two cell types after incubation for 24 h or 48 h. HepG2 cells (**A**,**B**), HepG2/ADM cells (**C**,**D**); 24 h (**A**,**C**); 48 h (**B**,**D**). *p < 0.05, **p < 0.01 compared with the controls; ^**#**^p < 0.05, ^**##**^p < 0.01 compared with the Dox group (*n* = 3).

The cytotoxicity of different Dox formulations toward resistant and sensitive cells was determined by MTT assay after incubation for 24 or 48 h. As shown in Fig. 8, blank-micelle hardly decreased the viability of HepG2 and HepG2/ADM cells even at a relatively high concentration. In HepG2 cells, the IC_50_ value of Dox was lower than that of Dox-micelle at 24 h (Fig. 8A), as Dox was directly located in the nucleus, whereas the release of loaded drug in Dox-micelle required a specific time period. This assumption was verified by the result of cytotoxicity at 48 h in HepG2 cells (Fig. 8B), as the IC_50_ values of Dox and Dox-micelle were much closer in 48 h than that in 24 h, resulting from more drugs released from Dox-micelle. On the contrary, Dox-micelle induced higher cytotoxicity in HepG2/ADM cells than Dox (Fig. 8C,D), which was due to the fact that P-gp, overexpressed in HepG2/ADM cells, was effluxed of drugs from intracellular to extracellular environments. Moreover, Dox-micelle could bypass the P-gp-mediated drug efflux under the advantages of micelles. Dox–siRNA-micelle led to further increases in cytotoxicity in HepG2/ADM cells, and improved the antitumor activity in HepG2/ADM cells close to that in HepG2 cells, which was associated with the siRNA-mediated decrease in drug efflux. Thus, Dox–siRNA-micelle could improve the therapeutic efficacy by bypassing drug efflux and downregulating the P-gp level.

### Intracellular accumulation of Dox

**Figure 9:**
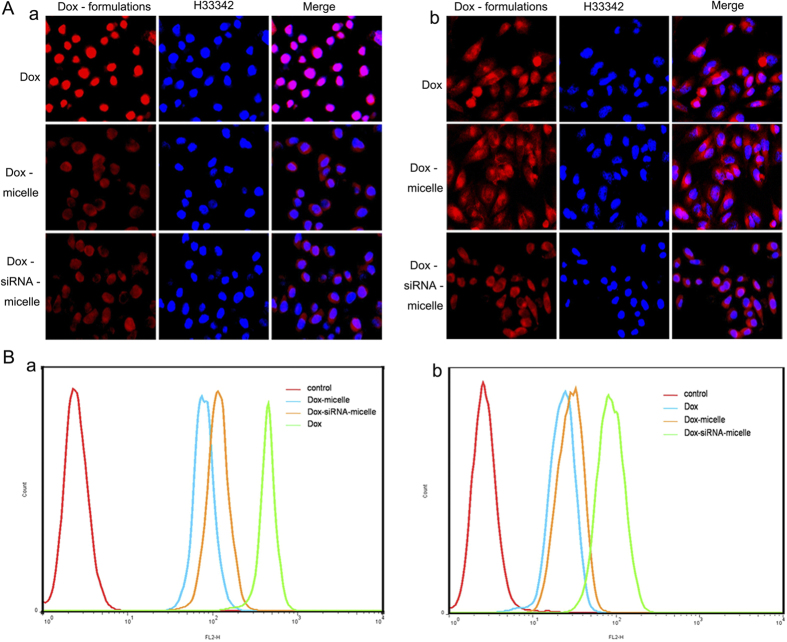
Comparison of intracellular Dox accumulation among HepG2 and HepG2/ADR cells after treatment with different Dox formulations. HepG2 cells (**A**), HepG2/ADM cells (**B**).

Intracellular accumulation and subcellular localization of the drug after incubation with different Dox formulations were detected by CLSM (Fig. 9A) and flow cytometry (Fig. 9B). The Dox fluorescence intensity in HepG2 cells (a) was stronger than that in HepG2/ADM cells (b) after treatment with free Dox, indicating that P-gp, which was overexpressed in HepG2/ADM cells, could pump out the intracellular drugs. Dox delivery by Dox-micelle resulted in higher fluorescence intensity in HepG2/ADM cells, which was attributed to bypassing the drug efflux under the help of micelles. Further increases in fluorescence intensity were observed after HepG2/ADM cells were incubated with Dox–siRNA-micelle; hence, downregulation of P-gp resulted in high intracellular drug accumulation. Results of quantitative determination of intracellular drug concentration in HepG2/ADM cells via flow cytometry were consistent with the CLSM results. These findings further confirmed the effectiveness of Dox–siRNA-micelle in improving therapeutic efficacy by suppressing P-gp expression and increasing intracellular Dox accumulation.

### Micelle distribution in nude mice

**Figure 10:**
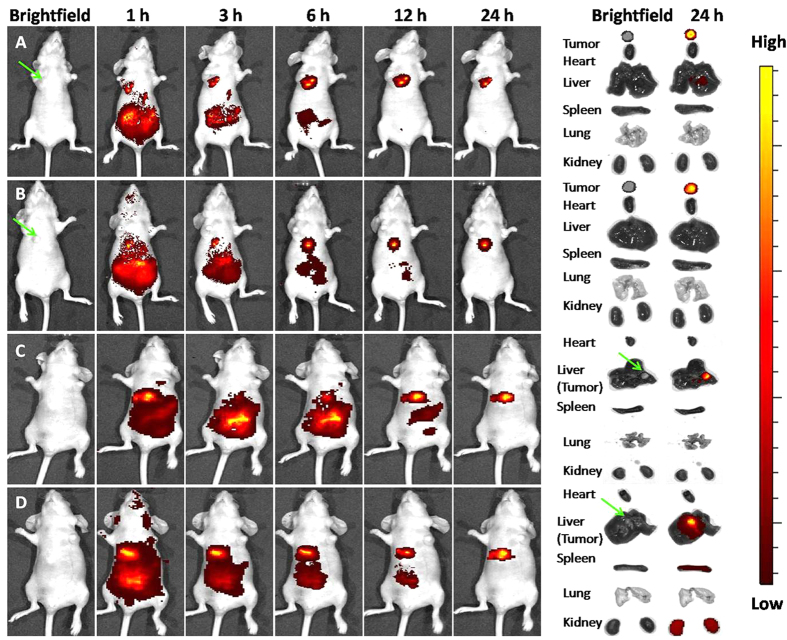
*In vivo* NIR fluorescence real-time imaging of subcutaneously transplanted HepG2 tumor (**A**) and HepG2/ADM tumor (**B**), as well as nude mice bearing HepG2 cells (**C**) and HepG2/ADM cells (**D**) *in situ* after i.v. of Cy-7-labeled micelle nanoparticles.

The potential targeting efficacy of micelles was evaluated by investigating the biodistribution of NIR fluorescence. The time-dependent fluorescence intensity was imaged from 1 h to 24 h post-administration (Fig. 10). After 24 h post-injection, almost all the fluorescent signals were located in the tumor region for the two types of tumor models, thereby indicating that micelles for co-delivery could circulate in the blood and accumulate in the tumor. That is to say, NSC, the hydrophilic shell, enables micelles to be stealthy and long circulating^9^, and micellar nanocarriers with an appropriate particle size of approximately 50–200 nm exhibit better tumor accumulation via the EPR effect^14^. The fluorescence of tumor and major organs after 24 h post-administration also indicated the effectiveness of micelles in tumor-targeted co-delivery and prevention of quick elimination by the liver and kidney.

### *In vivo* antitumor activity

**Figure 11:**
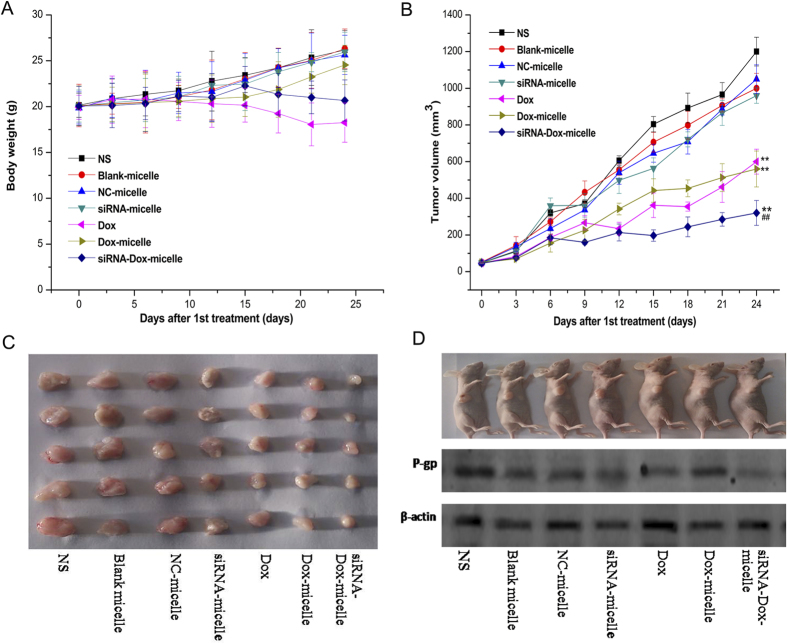
(**A**) Body weights of tumor-bearing nude mice in all groups (*n* = 5). (**B**) Antitumor effect of different formulations through i.v. injection (*n* = 5). (**C**) Size of collected tumor in all groups. (**D**) Representative of tumor-bearing nude mice from each group and its P-gp protein content detected by Western blot. **p < 0.01 compared with the NS group; ^**##**^p < 0.01 compared with the Dox group (*n* = 3).

Tumor suppression with various formulations administered *in vivo* was analyzed to evaluate the synergistic antitumor activity of siRNA–Dox-micelle. As shown in Fig. 11B, simultaneous delivery of siRNA and Dox by siRNA–Dox-micelle exhibited the strongest anti-tumor activity among the formulations. Furthermore, the synergistic antitumor activity of siRNA–Dox-micelle was demonstrated compared with siRNA-micelle and Dox-micelle treatments^28^,^37^. The delivery of Dox by Dox-micelle did not significantly increase tumor suppression compared with Dox only treatment, but significantly improved the survival quality of tumor-bearing mice, as the mice weight significantly decreased after Dox injection (Fig. 11A), indicating the safety of the micelle material. The size of the collected tumor was in accordance with the above results, and further confirmed the synergistic antitumor activity of siRNA–Dox-micelle (Fig. 11C). Analysis of P-gp expression in each treatment showed a consistent knockdown efficiency (Fig. 11D). The delivery of siRNA by siRNA-micelle could significantly downregulate P-gp expression for MDR reversal *in vivo*. The combination of Dox and siRNA-P-gp could enhance the chemotherapeutics and exert better tumor suppressive effects.

## Conclusions

Distinctive triblock polymer micelle was designed for co-delivery of Dox and siRNA–P-gp (Dox–siRNA-micelle). The prepared Dox–siRNA-micelle exhibited an average particle size of approximately 170 nm and instability at low pH, thereby enhancing tumor accumulation and intracellular release of the encapsulated drug and siRNA. Dox–siRNA-micelle could improve the therapeutic efficacy *in vitro* and *in vivo* by bypassing the drug efflux transporters, downregulating P-gp expression, and increasing intracellular Dox concentration. This study demonstrated the effectiveness of Dox–siRNA-micelle for tumor-targeted delivery, MDR reversal, and antitumor activity, and provided an effective strategy for the treatment of cancers that develop MDR.

## Additional Information

**How to cite this article**: Zhang, C.-g. *et al*. Novel polymer micelle mediated co-delivery of doxorubicin and P-glycoprotein siRNA for reversal of multidrug resistance and synergistic tumor therapy. *Sci. Rep.* **6**, 23859; doi: 10.1038/srep23859 (2016).

## Figures and Tables

**Figure 1:**
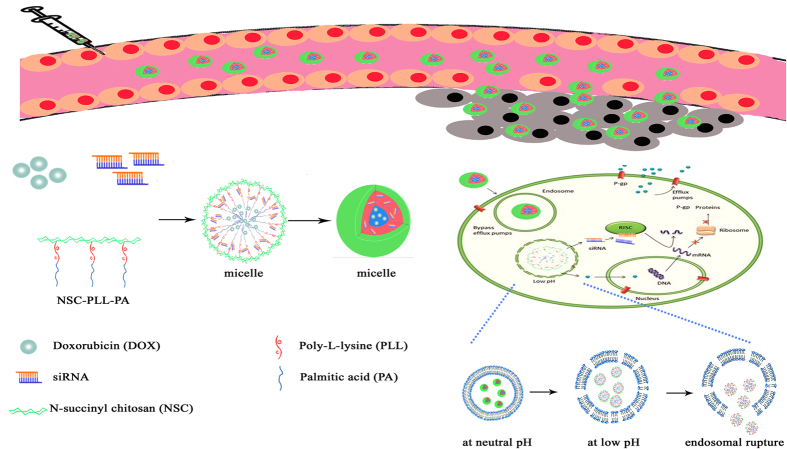
Schematic illustrating the mechanism of micelles for tumor-targeted delivery and synergistic tumor therapy.
